# Supplementary material for: Life history predicts global population responses to the weather in terrestrial mammals
Source: eLife. 2022 Jul 1;11:e74161. doi: 10.7554/eLife.74161 (PMC9307275; doi:10.7554/eLife.74161)
Supplement: Supplementary file 1. [file elife-74161-supp1.docx]

**
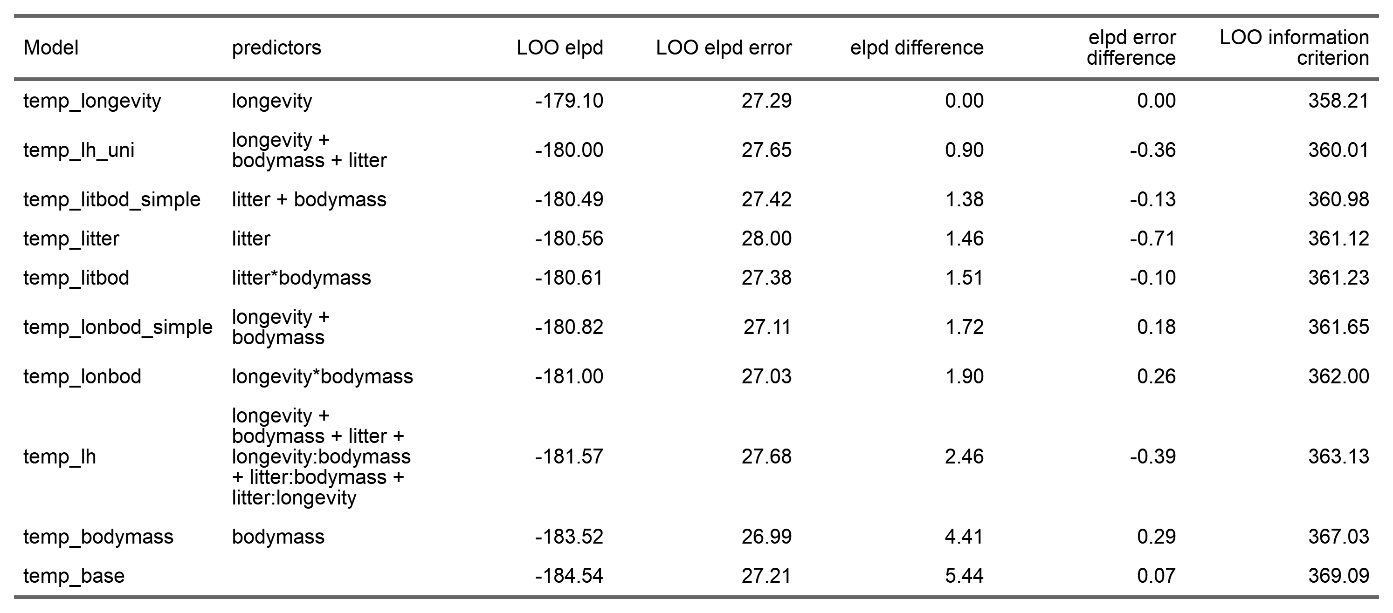
Supplementary File 1A: Table S1. Model selection results from Gamma models exploring the impact of life-history traits on absolute responses to temperature in the terrestrial mammals.** Leave One Out (LOO) cross validation results for 10 candidate models including life-history traits relative to a base model excluding life-history traits. elpd = expected log pointwise predictive density.

**Supplementary File 1B:
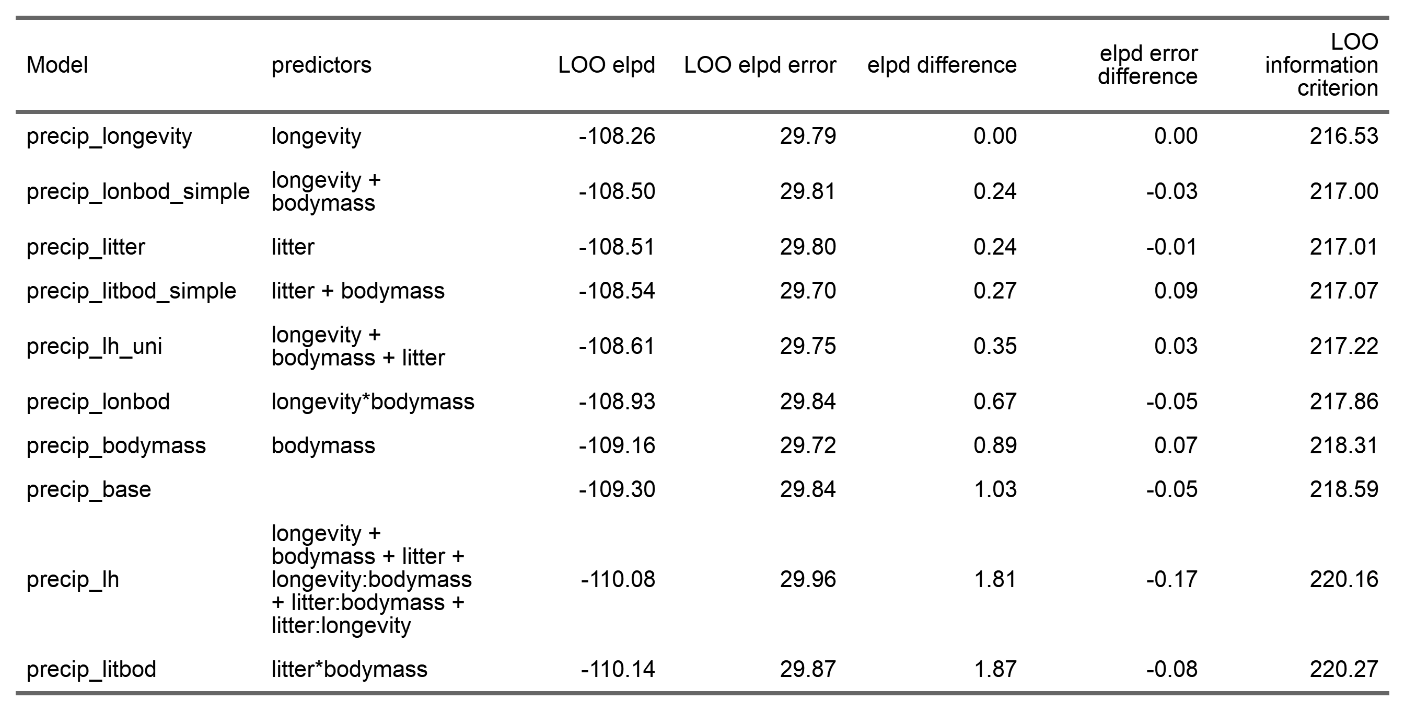
Table S2. Model selection results from Gamma models exploring the impact of life-history traits on absolute responses to precipitation in the terrestrial mammals.** Leave One Out (LOO) cross validation results for 10 candidate models including life-history traits relative to a base model excluding life-history traits. elpd = expected log pointwise predictive density.
