# Supplementary material for: Life history predicts global population responses to the weather in terrestrial mammals
Source: eLife. 2022 Jul 1;11:e74161. doi: 10.7554/eLife.74161 (PMC9307275; doi:10.7554/eLife.74161)
Supplement: Transparent reporting form [file elife-74161-transrepform1.pdf]

Westbrook Centre
Milton Road
Cambridge CB4 1YG, UK

P 01223 855340
W elifesciences.org
T @elife

eLife's transparent reporting form

We encourage authors to provide detailed information *within their submission* to facilitate the interpretation and replication of experiments. Authors can upload supporting documentation to indicate the use of appropriate reporting guidelines for health-related research (see EQUATOR Network), life science research (see the BioSharing Information Resource), or the ARRIVE guidelines for reporting work involving animal research. Where applicable, authors should refer to any relevant reporting standards documents in this form.

If you have any questions, please consult our Journal Policies and/or contact us: editorial@elifesciences.org.

Sample-size estimation

- You should state whether an appropriate sample size was computed when the study was being designed
- You should state the statistical method of sample size computation and any required assumptions
- If no explicit power analysis was used, you should describe how you decided what sample (replicate) size (number) to use

Please outline where this information can be found within the submission (e.g., sections or figure legends), or explain why this information doesn't apply to your submission:

This was a comparative study that extracted previously available data from a global database of vertebrate abundances. The total number of records for which we had sufficient abundance information was 486 from 157 species. We were thus limited i.e. could not select sample size, which was instead restricted based on our selection criteria. Instead, we used a Bayesian statistical framework with conservative, regularising priors that resulted in posteriors that were tuned to uncertainties presented by sample size restrictions.

Replicates

- You should report how often each experiment was performed
- You should include a definition of biological versus technical replication
- The data obtained should be provided and sufficient information should be provided to indicate the number of independent biological and/or technical replicates
- If you encountered any outliers, you should describe how these were handled
- Criteria for exclusion/inclusion of data should be clearly stated
- High-throughput sequence data should be uploaded before submission, with a private link for reviewers provided (these are available from both GEO and ArrayExpress)

Please outline where this information can be found within the submission (e.g., sections or figure legends), or explain why this information doesn't apply to your submission:

Replicates in the context of this comparative study occurred with repeated records for species. The number of records for each species ranged from 1-17, with a mean of 3.1 and median of 2 records per species. These replicates were again limited by the availability of data within the global database. We explicitly explored the role of within vs. among species variance and its implications on our findings. See Figure 2 and Results.

eLife Sciences Publications, Ltd is a limited liability non-profit non-stock corporation incorporated in the State of Delaware, USA, with company number 5030732, and is registered in the UK with company number FC030576 and branch number BR015634 at the address Westbrook Centre, Milton Road, Cambridge CB4 1YG.

Statistical reporting

- Statistical analysis methods should be described and justified
- Raw data should be presented in figures whenever informative to do so (typically when N per group is less than 10)
- For each experiment, you should identify the statistical tests used, exact values of N, definitions of center, methods of multiple test correction, and dispersion and precision measures (e.g., mean, median, SD, SEM, confidence intervals; and, for the major substantive results, a measure of effect size (e.g., Pearson's r, Cohen's d)
- Report exact p-values wherever possible alongside the summary statistics and 95% confidence intervals. These should be reported for all key questions and not only when the p-value is less than 0.05.

Please outline where this information can be found within the submission (e.g., sections or figure legends), or explain why this information doesn't apply to your submission:

> Statistical approaches, their justification, and comparisons between different statistical approaches are explained in full in Materials and Methods. Full analysis code, and explanations are given in the online Zenodo repository: 10.5281/zenodo.4707232. The approach for the meta-regression (core statistical technique) was a Bayesian hierarchical modelling framework and the use of Leave-One-Out Cross-validation. There are no frequentist statistics presented. Instead, we present model estimates and posterior prediction intervals to give the uncertainty of estimates, assess the convergence behaviour of Markov Chains, and assess model predictive performance with the LOO criterion of expected log wise predictive density, which is presented in Supplementary File 1 for our key findings of life-history effects.

(For large datasets, or papers with a very large number of statistical tests, you may upload a single table file with tests, Ns, etc., with reference to sections in the manuscript.)

Group allocation

- Indicate how samples were allocated into experimental groups (in the case of clinical studies, please specify allocation to treatment method); if randomization was used, please also state if restricted randomization was applied
- Indicate if masking was used during group allocation, data collection and/or data analysis

Please outline where this information can be found within the submission (e.g., sections or figure legends), or explain why this information doesn't apply to your submission:

> This is not appropriate for the current study, which was a comparative meta-regression based on previously collected data.

Additional data files ("source data")

- We encourage you to upload relevant additional data files, such as numerical data that are represented as a graph in a figure, or as a summary table
- Where provided, these should be in the most useful format, and they can be uploaded as "Source data" files linked to a main figure or table
- Include model definition files including the full list of parameters used
- Include code used for data analysis (e.g., R, MatLab)
- Avoid stating that data files are "available upon request"

Please indicate the figures or tables for which source data files have been provided:

All analysis, code, and data supporting the study is presented in full with extensive descriptions in the following Zenodo repository: 10.5281/zenodo.4707232. This repository enables all analyses to be fully reproducible given the public access of the necessary data. This repository includes all necessary data to produce the manuscript figures (see the data/ sub folder of the repository).

eLife Sciences Publications, Ltd is a limited liability non-profit non-stock corporation incorporated in the State of Delaware, USA, with company number 5030732, and is registered in the UK with company number FC030576 and branch number BR015634 at the address Westbrook Centre, Milton Road, Cambridge CB4 1YG.
